# Resting-state brain activity in Chinese boys with low functioning autism spectrum disorder

**DOI:** 10.1186/s12991-018-0217-z

**Published:** 2018-11-14

**Authors:** Gaizhi Li, Kathryn Rossbach, Wenqing Jiang, Yasong Du

**Affiliations:** 1 Shanxi Medical University, The First Hospital of Shanxi Medical University, Taiyuan, China; 2 NCSP, Greater Atlanta area, Atlanta, USA; 3 Department of Child & Adolescent Psychiatry, Shanghai Mental Health Center, Shanghai Jiao Tong University School of Medicine, No 600 Wanping Nan Road, Xuhui, Shanghai, 200030 China

**Keywords:** Low functioning autism spectrum disorder, Resting-state functional magnetic resonance imaging, Regional homogeneity, Amplitude of low-frequency fluctuation, Autism behavior checklist

## Abstract

**Background:**

This study aimed to explore the resting-state fMRI changes in Chinese boys with low functioning autism spectrum disorder (LFASD) and the correlation with clinical symptoms.

**Methods:**

The current study acquired resting-state fMRI data from 15 Chinese boys with LFASD and 15 typically developing (TD) boys to examine the local brain activity using the regional homogeneity (ReHo) and amplitude of low-frequency fluctuation (ALFF) indexes; the researchers also examined these measures and their possible relationships with clinical symptoms using the autism behavior checklist.

**Results:**

Results indicated that boys with LFASD exhibited increased ReHo in the right precuneus and inferior parietal gyrus (IPG), increased ALFF in right middle temporal gyrus, angular gyrus and IPG. However, no correlation was found between the ALFF/ReHo score and clinical symptoms in the LFASD group.

**Conclusions:**

Some of the brain regions had ReHo/ALFF values that were higher in the boys with LFASD than the TD group and these differentiated brain areas in boys with LFASD were all on the right cerebrum, which supported ‘atypical rightward asymmetry’ in boys with LFASD.

## Introduction

Autism spectrum disorders (ASDs) are increasingly prevalent neurodevelopmental disorders characterized by impaired social interaction and repetitive behaviors [1]. Long distance under-connectivity and local over-connectivity in individuals with autism have been reported by many studies [2, 3]. Research indicates that the regional connectivity differences found between samples with ASD and typically developing (TD) control groups should be examined carefully as high variability among ASD subjects may be contributing to these apparent differences.

Resting-state fMRI is a method of functional brain imaging that can be used to evaluate regional interactions that occur when a subject is not performing an explicit task [4]. Resting-state data obtained through the scans can be analyzed using a variety of methods. ReHo and ALFF are two methods widely used for characterizing local spontaneous activity of RS-fMRI data. ReHo measures the local synchronization of the time series of neighboring voxels, whereas ALFF/fALFF measures the amplitude of time series fluctuations at each voxel [5–7].

Many studies have been completed examining psychiatric disorders using ReHo and ALFF [8, 9], some of which studied ASD. Using the ReHo, both increases and decreases of ReHo value were observed in resting-state fMRI studies of individuals with ASD, but with poor replication across studies. For example, Paakki et al. [10] used the ReHo approach to study adolescents with ASD and found that compared with the controls, the subjects with ASD had significantly decreased ReHo in the right superior temporal sulcus region, right inferior and middle frontal gyri, right insula and right postcentral gyrus. Further, significantly increased ReHo was shown in the left inferior frontal and anterior subcallosal gyrus [10]. While Shukla et al. [11] found that ReHo was lower in the ASD than the TD group in the superior parietal and anterior prefrontal regions, higher ReHo was detected in lateral and medial temporal regions, predominantly in the right hemisphere in the ASD group, thus they proposed that ReHo is a sensitive measure for detecting cortical abnormalities in ASD. With regard to ALFF/fALFF, Itahashi et al. [12] reported that patients with High Functioning ASD showed significantly decreased fALFF values in a large cluster, including the right LING (lingual gyri), middle occipital gyrus (MOG), FG (fusiform gyri), and cerebellum. Di Martino et al. [13] used ReHo, ALFF, voxel mirrored homotopic connectivity (VMHC) and degree centrality (DC) in their research, and reported that two clusters exhibited ASD-related abnormalities in three measures, one from the left posterior insula to the central and parietal operculum (which exhibited ASD-related decreases in VMHC, ReHo and DC), and the other cluster was located in right dorsal superior frontal cortex (which exhibited ASD-related increases in fALFF, ReHo and DC).

These studies focused on high functioning autism spectrum disorder or Asperger syndrome, even though one-third of individuals with ASD are on the lower functioning end of the autism spectrum according to the latest CDC reports [14]. To our knowledge, no report of the correlation between clinical symptoms and ALFF/ReHo value has been examined in Chinese children with LFASD.

ASD is about four times more common among boys than among girls [14]; thus, it is more difficult to recruit girls for a study of this nature simply due to the lack of girls with ASD. Further, researchers proposed that the neurobiological mechanism for boys and girls may differ [15]. Therefore, results may be biased or inaccurate if a study was to include both boys and girls.

In the current research study, we aimed to explore the difference between the boys with LFASD and TD using Reho and ALFF, and examined the correlation between ReHo/ALFF and clinical symptoms. We hypothesized that (1) the ReHo, ALFF of resting-state brain activity would be different between boys with LFASD and typically developing controls in brain areas shown to display functional alterations in previous studies; (2) the differentiated brain areas may be correlated with the clinical symptoms.

## Methods

### Participants

Participants in the LFASD sample included a clinically referred sample of consecutive cases of children with an ASD diagnosis within the Shanghai Mental Health Center, Shanghai Jiao Tong University School of Medicine between April 2015 and April 2017. Diagnoses were based on DSM-5 (American Psychiatric Association, 2013) criteria and determined by an MD-level clinician, under the supervision of an MD/Ph.D. Professor (Dr. Du, the corresponding author). All participants also completed the WISC-IV Chinese version.

The children in the typically developing control group (TD group) were recruited from primary and middle schools in Shanghai. Children were excluded if they had any type of psychiatric disorder based on the Kiddie-SADS-present and lifetime version [16], which was conducted by an MD-level clinician, under the supervision of an MD/Ph.D. Professor (Dr. Du).

Parents of all the children completed the autism behavior checklist (ABC) after receiving instructions from the psychological professionals working in Shanghai Mental Health Center, Shanghai Jiao Tong University School of Medicine, and questionnaires were scored and interpreted by an MD-level clinician working in the Shanghai Mental Health Center, Shanghai Jiao Tong University School of Medicine. The ABC was used to rule out the presence of ASD-related symptoms in the TD group. No children in the control group had elevated scores on the ABC.

This study was approved by the Shanghai Mental Health Center Ethics Committee. Parents provided written informed consent for their children. Children who were verbal and could write their name, and also judged to be capable of providing assent, signed their name on this form too.

### Measures

#### WISC-IV Chinese version

The WISC-IV Chinese version is an individually administered instrument designed to measure intelligence [17]. The WISC-IV Chinese version contains 10 core subtests and five additional subtests. These are combined into four index scores [Verbal Comprehension Index (VCI), Perceptual Reasoning Index (PRI), Processing Speed Index (PSI), Working Memory Index (WMI)] and one full scale intelligence (FSIQ) score, which ranges from 40 to 160.

#### Autism behavior checklist

The autism behavior checklist includes 57 items and five domains, including the sensory, relating, body concept, language and social self-help domain [18]. It was introduced into China by Yang et al. and has been widely used in clinical and scientific research [19, 20]. It can be used with individuals aged from 18 months to 35 years, as a screening tool for ASD.

### MRI data acquisition

All MRI data were acquired using a 3.0 T MRI system (Siemens MAGNETOM Trio Tim) with a phased array whole-head coil. The functional images were acquired using a gradient echo-planar imaging sequence (repetition time (TR) 2000 ms, echo time (TE) 30 ms, flip angle 77°, slice thickness 3 mm, matrix size 74 * 74). Two-hundred and forty volumes were acquired in a single run.

During the scan, each of the participants was instructed to remain relaxed in the scanner, lying as still as possible, with his or her eyes closed, but to stay awake while in the dim scanner room. In addition, a high-resolution T1-weighted spoiled gradient recalled (SPGR) 3D MRI image was acquired (TR 2530 ms, TE 3.25 ms, 1-mm-slice thickness, matrix size 256 * 256).

### Data preprocessing

Preprocessing was performed in MATLAB R2010b (Mathworks, Natick, MA) using data processing assistant for resting-state fMRI (DPARSF advanced edition) software [21]. DPARSF pipeline analysis was used to do the preprocessing. The first step was to remove the first ten time points for signal equilibrium and to allow the participants’ adaptation to the scanning noise, slice timing and realign, then a report of head motion was created based on the realign parameters estimated by SPM (as shown in “ExcludeSubjects.txt” in the “Realign Parameter” directory). According to the report, three participants with ASD and three TD participants were excluded due to excessive motion (≥ ± 2.5 mm translation and ≥ ± 2.5 rotation from the first volume in any axis). For inter-subject comparison to be feasible, the individual brain was transformed or spatially normalized into a standardized template using unified segmentation on a T1 image. The whole-brain signal, six motion parameters, the cerebrospinal fluid (CSF), and the white matter signals were also removed as nuisance variables to reduce the effects of head motion and non-neuronal BOLD fluctuations in a regression analysis. A Gaussian filter (a 4 mm FWHM) was used to smooth the fMRI data (for ReHo, this step was performed after ReHo calculation). Removing the systematic drift or trend using a linear model was the last step.

### Statistical analysis

Resting-state fMRI data analysis toolkit (REST 1.8) was used to perform the data analysis [22]. To explore the between-group patterns, two-sample *t* tests were performed on the ALFF and ReHo, respectively, with age/FSIQ as covariates. Partial correlation analysis was applied to analyze the correlation between ALFF/ReHo and clinical symptoms using age/FSIQ as covariates in REST. A correction for multiple comparisons was performed using Monte Carlo simulation with a corrected threshold of *P *< 0.005 (two-tailed).

## Results

### Demographic data

Nineteen boys with LFASD participated in our study. One of them could not cooperate in the WISC-IV administration, so only 18 boys with LFASD completed the WISC-IV Chinese version, and three boys were excluded due to excessive head motion. The mean age of the LFASD group was 8.87 ± 3.11. Eighteen typically developing boys were included, three of whom were excluded due to excessive head motion; all the boys in the control group completed the WISC-IV Chinese version, and the mean age of the TD group was 10.53 ± 2.61. No significant difference was observed when it came to age between the two groups (t = − 1.588, *P *= 0.124). The mean FSIQ score was 50.47 ± 11.25 for the LFASD group and 127.27 ± 13.84 for the TD group, and the difference between the groups was statistically significant (*P *< 0.001). The clinical data of the LFASD group were assessed using the ABC and the total score is 66.50 ± 23.61; the subscale data are shown in Table 1.

**Table 1:** Demographic data of the LFASD and TD groups

	LFASD group (*n* = 15)	TD group (*n* = 15)	*t*	*P*
Age	8.87 ± 3.11	10.53 ± 2.61	− 1.588	0.124
FSIQ	50.47 ± 11.25	127.27 ± 13.84	− 16.679	< 0.001
ABC				
Sensory	12.94 ± 6.86	–		
Relating	12.81 ± 9.10	–		
Body concept	11.88 ± 6.84	–		
Language	15.94 ± 7.49	–		
Social self-help	12.94 ± 4.81	–		
Total ABC score	66.50 ± 23.61	–		

### Group difference

**Fig. 1:**
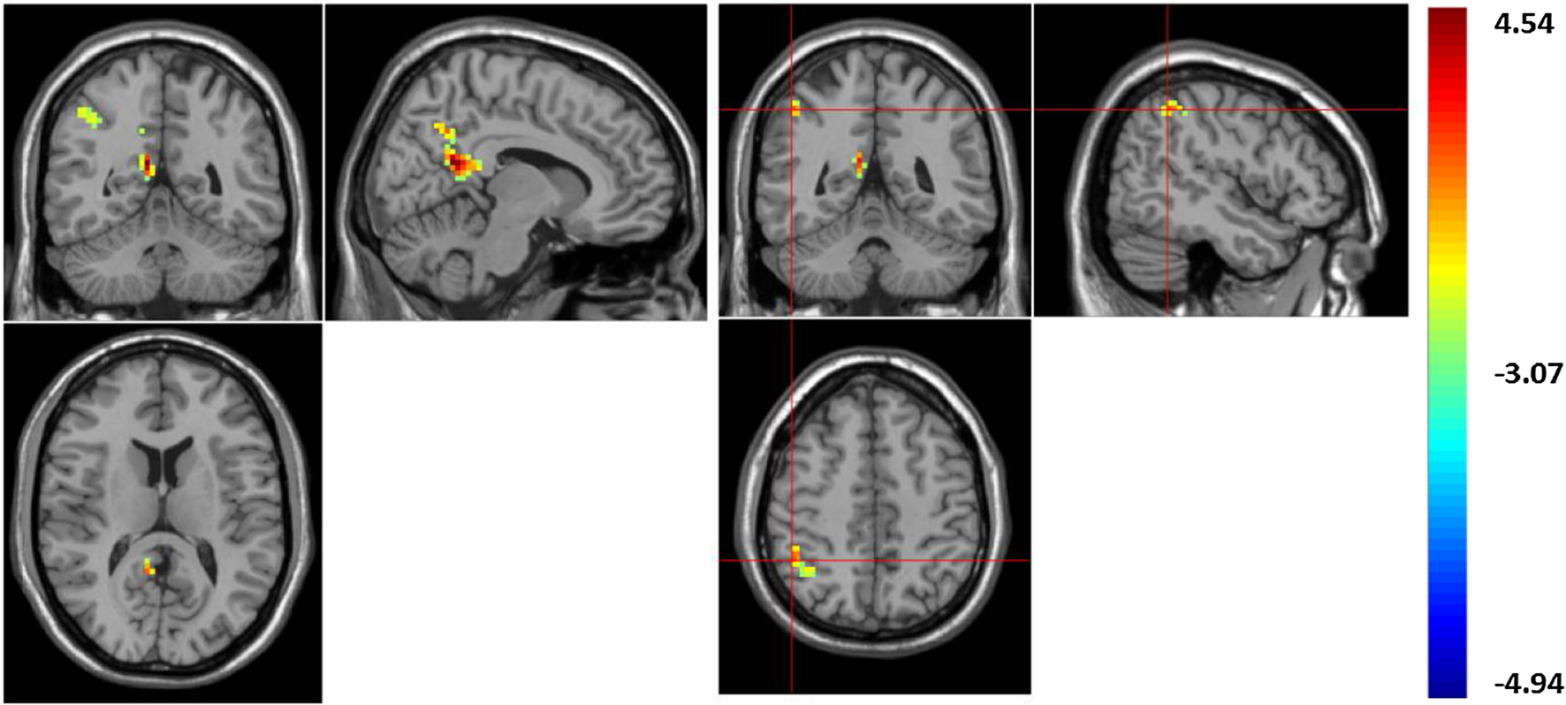
The brain area of increase ReHo value. ReHo value increased in brain areas: right precuneus; right inferior parietal gyrus (IPG)

**Fig. 2:**
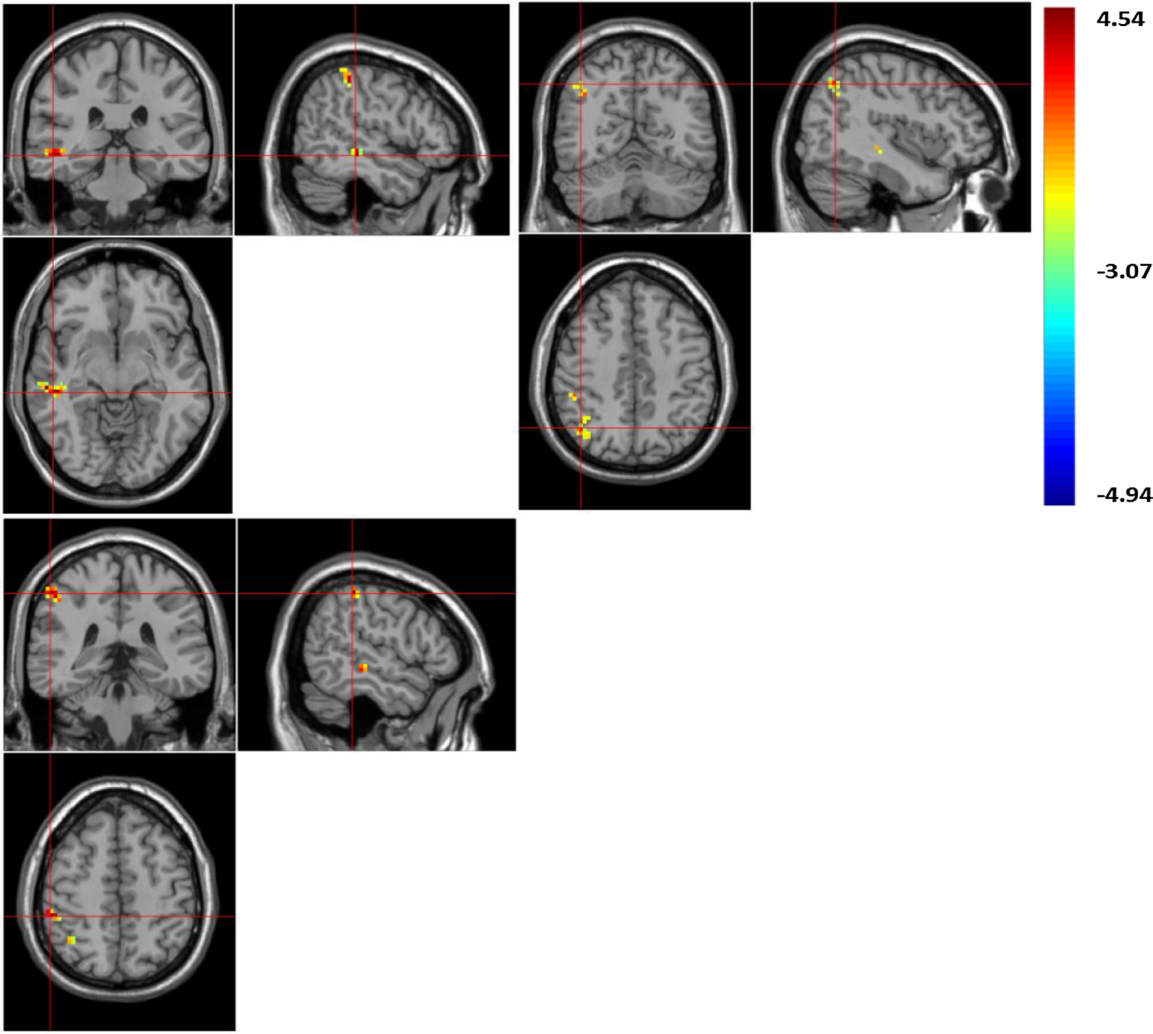
The brain area of increased ALFF value. ALFF value increased in brain areas: right middle temporal gyrus (MTG); right angular gyrus; right inferior parietal gyrus (IPG)

In the current research study, using ReHo values, the boys with LFASD had statistically significantly higher scores than the TD children when it came to the Precuneus (BA 23) and Inferior Parietal Gyrus, IPG (BA 40) (See Fig. 1 and Table 2). At the same time, using ALFF values, the boys with LFASD had statistically significantly higher scores when it came to the Right Middle Temporal Gyrus MTG (BA 21), Angular gyrus (BA 39) and IPG (BA 40) (see Fig. 2 and Table 2).

**Table 2:** The brain regions of ALFF/ReHo score between the LFASD and TD groups

	Voxels	L/R	Brain regions	BA	*x*	*y*	*z*	Peak
ALFF	22	R	MTG	21	51	− 30	− 9	4.4535
	36	R	Angular	39	42	− 63	45	4.0839
	22	R	IPG	40	54	− 36	51	4.5399
ReHo	59	R	Precuneus	23	9	− 51	18	4.4852
	35	R	IPG	40	48	− 48	48	3.8732

*MTG* middle temporal gyrus, *IPG* inferior parietal gyrus

### Correlation analysis

When examining the LFASD group, based on the correlation analysis between the ReHo value and the ABC subscale and total scores, no significant correlation was observed. In addition, there was no significant correlation observed between the ALFF score and ABC scores (see Table 3).

**Table 3:** Correlation between ALFF/ReHo score and behavioral scores (ABC)

	Brian areas	Sensory	Social	Language	Body concept	Self-care	Total score
ALFF	Right MTG						
	*r*	− 0.049	0.244	0.392	0.252	0.404	0.320
	*P*	0.875	0.421	0.185	0.406	0.171	0.286
	Right angular						
	*r*	− 0.434	0.049	0.173	0.121	0.082	− 0.003
	*P*	0.139	0.874	0.572	0.693	0.790	0.992
	Right IPG						
	*r*	− 0.338	0.057	− 0.267	0.150	0.288	− 0.052
	*P*	0.258	0.852	0.378	0.624	0.341	0.865
ReHo	Right precuneus						
	*r*	− 0.375	− 0.359	− 0.259	− 0.060	0.309	− 0.254
	*P*	0.207	0.229	0.393	0.846	0.304	0.403
	Right IPG						
	*r*	− 0.024	0.005	− 0.412	− 0.158	0.014	− 0.160
	*P*	0.939	0.987	0.162	0.607	0.965	0.602

*MTG* middle temporal gyrus, *IPG* inferior parietal gyrus

## Discussion

ASD is a neurodevelopmental disorder with an unknown biological basis. Many studies have confirmed the morphological difference between children with ASD and TD children [23–26]. As a part of a functional MRI study, resting-state data, especially the ReHo and ALFF, are two important methods widely used for characterizing local spontaneous brain activity of children with ASD. Up until now, evidence has been limited for children with low functioning ASD. In the current study, we explored the difference between Chinese boys with LFASD and TD boys using ReHo and ALFF. We also examined whether any relationship existed between abnormal spontaneous brain activity and clinical manifestation by looking at the pattern of relationships between regional brain measures and clinical symptoms in the LFASD group.

Unfortunately, excessive motion and floor task performance are issues that hinder successful scanning of young children and account for significant data loss [27] and, in our study, seven participants’ data had to be discarded due to head motion and lack of cooperation.

With respect to the group difference, our findings show that all the differentiated brain areas are on the right side of the cerebrum. These results within the right cerebrum are the same as reports from Shukla et al. [11] as they also found that higher ReHo were predominantly in the right hemisphere in the ASD group. Atypical rightward asymmetry was also reported to be a feature in ASD in another study [28].

According to our findings, there are four regions: precuneus (BA 23), IPG (BA 40), MTG (BA 21) and angular gyrus (BA 39) that significantly differ from typically developed children. MTG (BA 21) which is a part of temporal lobe has been regarded as an important brain area mainly engaged in the face recognition. Interestingly, the impairment of face recognition in individuals with ASD is frequently reported [29]. Cheng et al. [30] used rs-fMRI in a large sample to explore the functional connectivity of subjects with autism and controls. They identified two key systems, the first one in the middle temporal gyrus/superior temporal sulcus region that has reduced cortical functional connectivity (and increased with the medial thalamus). This area is implicated in face expression processing involved in social behavior. The middle temporal gyrus system is also implicated in theory of mind processing. A second key system is in the precuneus/superior parietal lobule region with reduced functional connectivity, which is implicated in spatial functions including of oneself and of the spatial environment. Our finding regarding the different resting state in boys with LFASD is partly consistent with Cheng’s study, as the precuneus/superior parietal lobule regions were both reported; however, the Angular gyrus was not observed in his study.

The precuneus (BA 23), IPG (BA 40) and angular gyrus (BA 39) are part of the occipital lobe. The precuneus (BA 23) is reported to be correlated with the reflective, self-related processing, awareness and conscious information processing, episodic memory, and visuospatial processing [31]. IPG (BA 40) has been involved in the perception of emotions in facial stimuli and interpretation of sensory information. The inferior parietal lobule is concerned with language, mathematical operations, and body image, particularly the supramarginal gyrus and the angular gyrus. The angular gyrus (BA 39) is engaged in transferring visual information to Wernicke’s area, in order to make meaning out of visually perceived words. It is also involved in a number of processes related to language, number processing and spatial cognition, memory retrieval, attention, and theory of mind [32].

Nonetheless, in the current study, no significant correlations were found for the ALFF/ReHo values and clinical symptoms. In past studies, facial recognition, spatial cognition and theory of mind were the basic impairments in resting time of children with ASD, but these neuro-psychological features were not assessed in the current study. This may be the reason that no relationship has been found between spontaneous brain activity and clinical manifestation. Therefore, the increased ALFF/ReHo may not be the neurological bases for clinical symptoms in the boys with LFASD. Thus, we suggest that the local connectivity of LFASD was higher than the TD group, but future larger samples will be needed to clarify this question.

The current results indicate that the resting-state activity is impaired mainly in the right hemisphere of the boys with LFASD, which is important for clinical practice. If results are able to be replicated, when boys with LFASD are being treated, the focus could be surrounding the right hemisphere. Additionally, this research could help support locating a more specific deficit in brain function related to ASD which could eventually aid in earlier detection and better treatment overall.

## Limitations

There are some limitations in our study. The first is that since we studied Chinese boys with LFASD, the sample size was relatively small. By only using boys in the study we cannot generalize our studies across genders. In addition, this is a cross-sectional study, thus we cannot conclude the developmental-related changes, even though age was as covariate when analyzed. Additionally, the typical developing group had a fairly high FSIQ; this was simply a result of the sample used, but may have impacted the study since the difference in IQ was so large between the two groups. Future longitudinal studies are needed to characterize the brain-clinical associations in Chinese boys with LFASD.

## Conclusion

In the present study, we used the measures of ReHo/ALFF to study the relationship between local connectivity and clinical symptoms in Chinese boys with LFASD. Some of the brain regions had ReHo/ALFF values that were higher in the boys with LFASD than the TD group. According to our study, all the resting-state differentiated brain areas in boys with LFASD were on the right cerebrum, which supported ‘atypical rightward asymmetry’ in boys with LFASD.

## Abbreviations

ABC: autism behavior checklist
ALFF: amplitude of low-frequency fluctuation
ASD: autism spectrum disorder
BA: Brodmann area
BOLD: blood oxygenation level dependent
CARS: Childhood Autism Rating Scale
DSM-5: diagnostic and statistical manual of mental disorders (5th ed)
FC: functional connectivity
FOV: field of view
FSIQ: full scale IQ
FWHM: full width half maximum
HFASD: high functioning autism spectrum disorder
IPG: inferior parietal gyrus
IQ: intelligence quotient
K-SADS-PL: Kiddie schedule for affective disorders and schizophrenia-present and lifetime version
LFASD: low functioning autism spectrum disorder
MNI: monireal neurological institute
MTG: middle temporal gyrus
NVIQ: nonverbal IQ
PRI: Perceptual Reasoning Index
PSI: Processing Speed Index
ReHo: regional homogeneity
ROI: region of interest
SNR: signal to noise ratio
TD: typically developing
TR/TE: repetition time/echo time
VCI: Verbal Comprehension Index
VIQ: verbal IQ
WISC-IV: Wechsler Intelligence Scale for children, the fourth edition
WMI: Working Memory Index
